# Efficient delivery of mesenchymal stem/stromal cells to injured liver by surface PEGylation

**DOI:** 10.1186/s13287-023-03446-w

**Published:** 2023-08-22

**Authors:** Yukiya Takayama, Kosuke Kusamori, Yuri Katsurada, Shu Obana, Shoko Itakura, Makiya Nishikawa

**Affiliations:** 1 Laboratory of Biopharmaceutics, Faculty of Pharmaceutical Sciences, Tokyo University of Science, 2641 Yamazaki, Noda, Chiba 278-8510 Japan; 2 Department of Pharmacy, Kobe City Hospital Organization, Kobe City Medical Center General Hospital, Chuo-Ku, Kobe, 650-0047 Japan; 3 Laboratory of Cellular Drug Discovery and Development, Faculty of Pharmaceutical Sciences, Tokyo University of Science, 2641 Yamazaki, Noda, Chiba 278-8510 Japan

**Keywords:** Cell surface modification, Liver failure, Lung entrapment, Mesenchymal stem/stromal cell, Polyethylene glycol

## Abstract

**Background:**

Mesenchymal stem/stromal cells (MSCs) have been used in clinical trials for various diseases. These have certain notable functions such as homing to inflammation sites, tissue repair, and immune regulation. In many pre-clinical studies, MSCs administered into peripheral veins demonstrated effective therapeutic outcomes. However, most of the intravenously administered MSCs were entrapped in the lung, and homing to target sites was less than 1%. This occurred mainly because of the adhesion of MSCs to vascular endothelial cells in the lung. To prevent this adhesion, we modified the surface of MSCs with polyethylene glycol (PEG; a biocompatible polymer) using the avidin–biotin complex (ABC) method.

**Methods:**

The surface of MSCs was modified with PEG using the ABC method. Then, the cell adhesion to mouse aortic endothelial cells and the tissue distribution of PEG-modified MSCs were evaluated. Moreover, the homing to the injured liver and therapeutic effect of PEG-modified MSCs were evaluated using carbon tetrachloride-induced acute liver failure model mice.

**Results:**

The PEG modification significantly suppressed the adhesion of MSCs to cultured mouse aortic endothelial cells as well as the entrapment of MSCs in the lungs after intravenous injection in mice. PEG-modified MSCs efficiently homed to the injured liver of carbon tetrachloride-induced acute liver failure model mice. More importantly, the cells significantly suppressed serum transaminase levels and leukocyte infiltration into the injured liver.

**Conclusion:**

These results indicate that PEG modification to the surface of MSCs can suppress the lung entrapment of intravenously administered MSCs and improve their homing to the injured liver.

**Graphical Abstract:**

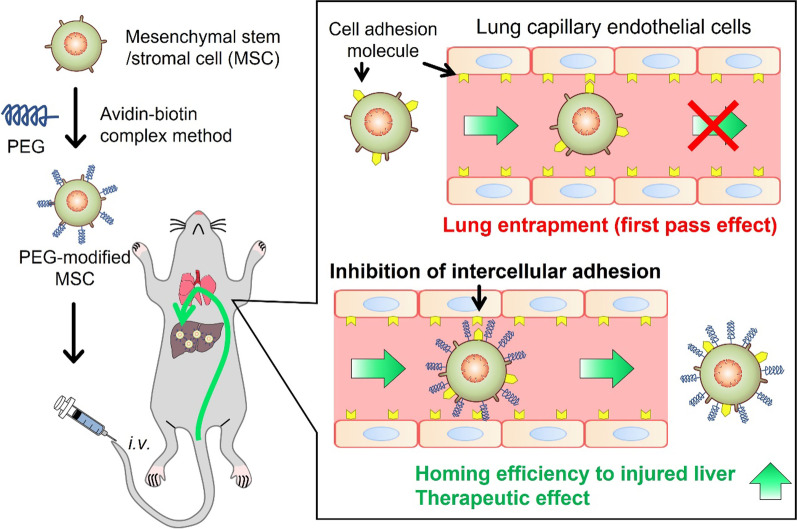

**Supplementary Information:**

The online version contains supplementary material available at 10.1186/s13287-023-03446-w.

## Background

Mesenchymal stem/stromal cells (MSCs) are multipotent stem/progenitor cells. These can be isolated from various tissues including the bone marrow, adipose tissue, and umbilical cord [1–5]. After systemic administration, MSCs migrate to ischemic and inflammatory sites and exhibit immune regulatory and tissue repair activities by secreting cytokines, growth factors, and extracellular vesicles, as well as by direct cell-to-cell interactions [6, 7]. Furthermore, the high expandability and low immunogenicity of the cells enable the administration of autologous MSCs as well as allogeneic MSCs [5]. The number of clinical trials of MSCs is exponentially increasing worldwide. Most of these trials focus on the treatment of cardiovascular diseases, graft-versus-host disease, immune system diseases, wounds, and tissue restoration [8]. In addition, certain MSC products have been approved, including Prochymal and TEMCELL HS [9].

In many pre-clinical and clinical studies, MSCs are administered into peripheral veins because intravenous administration is a fundamental and minimally invasive administration method [10]. Furthermore, few adverse events have been reported in clinical studies in which MSCs were administered intravenously [10, 11]. However, the homing rate of intravenously administered MSCs to target sites was less than 1% [12]. This was mainly because of their entrapment in the lung vasculature [13]. It is considered that the therapeutic mechanism of MSCs involves both paracrine effects [14–16] and cell-to-cell interactions in injured sites [17, 18]. Therefore, it is necessary to prevent lung entrapment and improve homing efficiency to target sites for effective MSC-based therapy. A few studies have reported that the interaction between cell adhesion molecules expressed on MSCs and vascular endothelial cells in the lung plays an important role in the lung entrapment of intravenously administered MSCs [19, 20]. The inhibition of this interaction suppressed the lung entrapment of MSCs. Kerkelä et al. [21] reported that the decomposition of cell membrane proteins of MSCs by pronase significantly suppressed lung entrapment. Wang et al. [22] obtained similar results by blocking integrins expressed on MSCs using monoclonal antibodies. However, these approaches may affect the homing of MSCs to target sites and their therapeutic efficiency. This is because the functions of cell membrane proteins are closely related to homing and cell-to-cell interactions. Therefore, the development of alternative approaches to prevent lung entrapment without significantly influencing the characteristics of MSCs (except for cell adhesion) is highly desirable.

Polyethylene glycol (PEG) is a biocompatible polymer that has been used frequently in the research field pertaining to pharmaceutical sciences and cell engineering to improve the survival of transplanted cells by bypassing immune systems [23, 24]. Teramura et al*.* reported that cell surface modification with high molecular weight PEG (> 20 kDa; larger than the extracellular domains of cell adhesion molecules such as integrins) was effective for regulating the adhesion of L929 murine fibroblast cell line, human acute lymphoblastic leukemia T-cells, and human primary hepatocytes to scaffolds [25]. Therefore, PEG modification to the cell surface may be effective for preventing lung entrapment and improving the efficiency of homing to the target site of intravenously administered MSCs. However, conventional cell surface modification methods are generally toxic to MSCs because of unfavorable culture conditions in medium containing organic solvents for a long period [26]. To solve these disadvantages, we demonstrated earlier that cell surface modification using the avidin–biotin complex (ABC) method was effective for stable modification of MSCs with biotinylated and avidinated compounds in a short reaction time without significant modifications in the characteristics of MSCs [27, 28].

In this study, we modified the surface of MSCs with PEG using the ABC method and evaluated the effect of PEG modification on the lung entrapment and homing efficiency of MSCs to inflammation sites in mice. First, the surface of the murine mesenchymal stem cell line C3H10T1/2 cells was modified with PEG using the ABC method. Then, the influence of PEG modification on the characteristics of C3H10T1/2 cells was evaluated. Moreover, the cell adhesion to mouse aortic endothelial cells (MAECs) and the tissue distribution of intravenously administered PEG-modified C3H10T1/2 (PEG-C3H10T1/2) cells were evaluated. Finally, the homing to the injured liver and therapeutic effect of PEG-modified murine adipose-derived mesenchymal stem cell line m17.ASC (PEG-m17.ASC) cells were evaluated using carbon tetrachloride (CCl_4_)-induced acute liver failure model mice.

## Methods

### Materials

Isoflurane, streptavidin, 4% paraformaldehyde phosphate buffer solution, penicillin–streptomycin–l-glutamine solution, 0.4% trypan blue solution, sodium bicarbonate, Alizarin Red S (3,4-dihydroxy-9,10-dioxo-2-anthracenesulfonic acid sodium salt), Oil Red O (1-(2,5-dimethyl-4-(2,5-dimethylphenyl) phenyldiazenyl) azonapthalen-2-ol), Triton X-100, bovine serum albumin, carbon tetrachloride (CCl_4_), olive oil, and transaminase CII-test Wako kit were purchased from FUJIFILM Wako Pure Chemical Co. (Osaka, Japan). A trypsin–EDTA solution (0.25% trypsin and 1 mM EDTA) and antibiotic–antimycotic mixed stock solution (100×) were purchased from Nacalai Tesque Inc. (Kyoto, Japan). Dulbecco's modified Eagle's medium (DMEM) was purchased from Nissui Co., Ltd. (Tokyo, Japan). Claycomb medium, Medium 199, Hanks' balanced salt solution (HBSS), Alcian Blue staining solution, and carboxyfluorescein diacetate succinimidyl ester (CFSE) were purchased from Sigma-Aldrich Co. (St. Louis, MO, USA). Nunc Lab-Tek II Chamber Slides and Nunc Lab-Tek II Chambered Coverglasses were purchased from Thermo Fisher Scientific Inc. (Waltham, MA, USA). Fetal bovine serum (FBS) was purchased from Biosera (East Sussex, UK). Hygromycin B Gold was purchased from Invitrogen Life Technologies, Inc. (Carlsbad, CA, USA). Alexa Fluor 488 PEG Biotin (3400 Da, Alexa488-PEG-biotin) was purchased from Nanocs Inc. (New York, NY, USA). Anti-FAK (phospho Y397) antibody (anti-p-FAK IgG) was purchased from Abcam (Cambridge, UK). The O.C.T. compound was purchased from Sakura Finetechnical Co., Ltd. (Tokyo, Japan). Mesenchymal stem cell adipogenic differentiation medium, mesenchymal stem cell chondrogenic differentiation medium, and mesenchymal stem cell osteogenic differentiation medium were purchased from Clontech Laboratories Inc. (Mountain View, CA, USA). The Nano-Glo luciferase assay reagent was purchased from Promega Co. (Madison, WI, USA). Fluoromount-G was purchased from SouthernBiotech (Birmingham, AL, USA). VECTASHIELD Antifade Mounting Medium with DAPI was purchased from Vector Laboratories Inc. (Burlingame, CA, USA). Biotin-functionalized methoxy polyethylene glycol 2000 (PEG_2k_-biotin, 2000 Da) and biotin functionalized methoxy polyethylene glycol 20,000 (PEG_20k_-biotin, 20,000 Da) were purchased from Biopharma PEG Scientific Inc. (Watertown, MA, USA). All the other chemicals were of the highest commercially available grade.

### Cell culture

C3H10T1/2 cells were provided by Dr. Hiroki Kagawa (Department of Cell Biology, Kyoto Pharmaceutical University, Kyoto, Japan) and cultured in DMEM supplemented with 10% heat-inactivated FBS, 0.15% sodium bicarbonate, and 1% penicillin–streptomycin–l-glutamine solution. NanoLuc luciferase (Nluc)- and enhanced green fluorescent protein (GFP)-expressing C3H10T1/2 cells (C3H10T1/2/Nluc cells and C3H10T1/2/GFP cells, respectively) were cultured in DMEM supplemented with 10% heat-inactivated FBS, 0.15% sodium bicarbonate, 1% penicillin–streptomycin–l-glutamine solution, and 200 μg/mL hygromycin B. These cells were established in our previous reports [25]. m17.ASC cells were purchased from DS Pharma Biomedical (Osaka, Japan) and cultured in Claycomb medium supplemented with 10% heat-inactivated FBS and 1% penicillin–streptomycin-l-glutamine solution. Nluc-expressing m17.ASC (m17.ASC/Nluc) cells (which were established in our previous reports [26]) were cultured in Claycomb medium supplemented with 10% heat-inactivated FBS, 1% penicillin–streptomycin–l-glutamine solution, and 200 μg/mL hygromycin B. MAECs were provided by Professor Ichiro Saito (Department of Pathology, Tsurumi University School of Dental Medicine, Yokohama, Japan) and cultured in Medium 199 supplemented with 10% heat-inactivated FBS and 1% antibiotic–antimycotic mixed stock solution.

### Evaluation of cell surface modification with PEG

C3H10T1/2 cells (2 × 10^5^ cells) were seeded into 100 mm cell culture dishes and incubated in a CO_2_ incubator for 3 days. To modify the surface of C3H10T1/2 cells with PEG, C3H10T1/2 cells were detached from dishes using a trypsin–EDTA solution, washed two times with PBS, and collected in centrifuge tubes. The cells were then treated with 1 mM sulfo-NHS-LC-biotin for 20 min at room temperature, 50 μg/mL streptavidin for 10 min at 4 °C, and 100 μM PEG-biotin for 10 min at 4 °C. In each step, the cells were washed two times with PBS or HBSS. To verify the modification, streptavidin-modified C3H10T1/2 and unmodified C3H10T1/2 cells were incubated with 10 μM FITC-PEG-biotin for 10 min at 4 °C. After removing excess reagents by PBS wash, the cells were seeded onto Nunc Lab-Tek II Chamber Slide and incubated for 3 h in a CO_2_ incubator. After incubation, the cells were fixed with 4% paraformaldehyde solution and observed under a confocal laser scanning microscope (TCS SP8, Leica Microsystem, Mannheim, Germany). In addition, after the FITC-PEG-biotin treatment, the cells were lysed using cell lysis buffer M, and the amount of PEG modified on the cells was determined by measuring the fluorescence intensity at an Ex/Em wavelength of 485/535 nm using a microplate reader (Wallac 1420 ARVO, PerkinElmer, Inc., Waltham, MA, USA). To evaluate the retention time of PEG modification, streptavidin-modified C3H10T1/2 cells were incubated with 50 μM Alexa488-PEG-biotin and seeded onto Nunc Lab-Tek II Chambered Coverglasses (1 × 10^4^ cells/well). At 1, 3, and 6 h and 1, 2, 3, and 5 days after incubation, the cells were fixed with 4% paraformaldehyde solution and observed using a TCS SP8 confocal laser scanning microscope. In addition, Alexa488-PEG modified C3H10T1/2 cells were seeded into a 24-well culture plate, and the fluorescence intensity at an Ex/Em wavelength of 485/535 nm was measured using a Wallac 1420 ARVO microplate reader.

### Characteristics of PEG-MSCs

To evaluate the cytotoxicity of PEG modification, the viability of PEG_2k_-modified C3H10T1/2 (PEG_2k_-C3H10T1/2) cells and PEG_20k_-modified C3H10T1/2 (PEG_20k_-C3H10T1/2) cells was evaluated using the trypan blue exclusion assay immediately after modification. PEG_2k_-C3H10T1/2 cells, PEG_20k_-C3H10T1/2 cells, and unmodified C3H10T1/2 cells were seeded into a 6-well culture plate (5 × 10^4^ cells/well) to evaluate the cell proliferation of PEG-C3H10T1/2 cells. Furthermore, the number of cells was measured daily using the trypan blue exclusion assay. To evaluate cell differentiation, PEG_2k_-C3H10T1/2 cells, PEG_20k_-C3H10T1/2 cells, and unmodified C3H10T1/2 cells were cultured in adipogenic, osteogenic, or chondrogenic differentiation media according to the manufacturer's protocol. Furthermore, these were stained with Oil Red O, Alizarin Red S, and Alcian Blue staining solution, respectively, as reported previously [27].

### In vitro adhesion assay

PEG_2k_-C3H10T1/2/Nluc cells, PEG_20k_-C3H10T1/2/Nluc cells, and unmodified C3H10T1/2/Nluc cells (2 × 10^4^ cells/well) were seeded in a 96-well culture plate and incubated for 0.5, 1, 2, and 3 h in a CO_2_ incubator. Then, nonadherent cells were removed by washing two times with PBS, and the number of adherent cells was evaluated by measuring the luciferase activity of the lysed cells using a Wallac 1420 ARVO microplate reader. To evaluate the adhesion of PEG-C3H10T1/2 cells to vascular endothelial cells, MAECs (2.5 × 10^4^ cells/well) were seeded in a 96-well culture plate and incubated overnight in a CO_2_ incubator. PEG_2k_-C3H10T1/2/Nluc cells, PEG_20k_-C3H10T1/2/Nluc cells, and unmodified C3H10T1/2/Nluc cells (2 × 10^4^ cells/well) were seeded onto monolayered MAECs. Moreover, nonadherent C3H10T1/2/Nluc cells were removed by washing two times with PBS at 0.5, 1, 2, and 3 h after seeding. The number of C3H10T1/2/Nluc cells adhering to MAECs was determined by measuring the luciferase activity of the lysed cells. To observe PEG-C3H10T1/2 cells adhering to MAECs, MAECs (4 × 10^4^ cells/well) were seeded onto Nunc Lab-Tek II Chambered Coverglasses and incubated overnight in a CO_2_ incubator. PEG_2k_-C3H10T1/2/Nluc cells, PEG_20k_-C3H10T1/2/Nluc cells, and unmodified C3H10T1/2/Nluc cells (2 × 10^4^ cells/well) were seeded onto monolayered MAECs and incubated for 2 h in a CO_2_ incubator. Nonadherent C3H10T1/2/Nluc cells were removed by washing two times with PBS. Cells were fixed with 4% paraformaldehyde solution, mounted with VECTASHIELD Antifade Mounting Medium with DAPI, and observed using a digital microscope (BZ-9000, Keyence).

### Immunostaining

MAECs (4 × 10^4^ cells/well) were seeded onto Nunc Lab-Tek II Chambered Coverglasses and incubated overnight in a CO_2_ incubator. Then, PEG-modified or unmodified C3H10T1/2/GFP cells (1 × 10^4^ cells/well) were seeded onto monolayered MAECs. The culture medium was removed 2 h after incubation, and the cells were incubated with 4% paraformaldehyde solution. Thirty minutes after incubation, 0.2% Triton X-100 in PBS and 1% bovine serum albumin in PBS were added to achieve permeabilization and blocking, respectively. The fixed cells were then incubated with anti-phospho-focal adhesion kinase (p-FAK) IgG primary antibody for 1 h at room temperature and stained with tetramethylrhodamine isothiocyanate (TRITC)-labeled secondary antibody for 1 h at room temperature. After immunostaining, the samples were mounted with Fluoromount-G and observed using a TCS P8 confocal laser scanning microscope. The fluorescence intensity of phospho-FAK observed in stained C3H10T1/2/GFP cells was measured by region of interest (ROI) analysis. Then, the average pixel intensity (pixel intensity per cell) of 15–20 randomly selected C3H10T1/2/GFP cells was calculated.

### Animals

Male C3H/He mice (4–8 weeks-old) were purchased from Sankyo Labo Service Co., Inc. (Tokyo, Japan). Male FVB mice (4–8 weeks-old) were purchased from CLEA Japan Inc. (Tokyo, Japan). The mice were maintained under specific pathogen-free (SPF) conditions. All the animal experiments were conducted in accordance with the principles and procedures outlined in the National Institutes of Health Guide for the Care and Use of Laboratory Animals. The protocols for the animal experiments were approved by the Animal Experimentation Committee of the Tokyo University of Science. All the mice were euthanized by cervical dislocation under isoflurane anesthesia.

### Tissue distribution of PEG-MSCs

To evaluate the tissue distribution of intravenously injected PEG-MSCs, PEG_20k_-C3H10T1/2/Nluc cells or unmodified C3H10T1/2/Nluc cells (1 × 10^6^ cells/mouse in 100 μL of PBS) were injected into the tail vein of C3H/He mice under isoflurane anesthesia. This experiment was conducted using 6 mice (*n* = 3 PEG_20k_-C3H10T1/2/Nluc group versus *n* = 3 unmodified C3H10T1/2/Nluc group). An hour after injection, blood was collected from the inferior vena cava under isoflurane anesthesia, and the mice were euthanized by cervical dislocation. Then, the organs were collected. The luciferase activity in the lysates of tissues and whole blood was measured as reported previously [29]. In addition, to observe the cells in the lung, PEG-modified or unmodified C3H10T1/2 cells were labeled with CFSE as reported previously [30] and were injected into the tail vein of C3H/He mice under isoflurane anesthesia. An hour after injection, the lungs were excised from euthanized mice and fixed with 4% paraformaldehyde solution followed by embedding in O.C.T. compound. The lung samples were frozen using liquid nitrogen and sliced into 10-μm-thick sections using a cryostat (CM3050 S, Leica Biosystems, Wetzlar, Germany). The tissue slides were observed under a BZ-9000 digital microscope. To evaluate the homing of MSCs to the injured liver, a CCl_4_-induced acute liver failure model was established by intraperitoneal injection of 1.5 mL/kg CCl_4_ dissolved in olive oil (1:1 ratio) into FVB mice. Six hours after CCl_4_ injection, PEG_20k_-m17.ASC/Nluc cells or unmodified m17.ASC/Nluc cells (1 × 10^6^ cells/mouse in 100 μL of PBS) were injected intravenously under isoflurane anesthesia. This experiment was conducted using 8 mice (*n* = 4 PEG_20k_-m17.ASC/Nluc group versus *n* = 4 unmodified m17.ASC/Nluc group). Twenty four hours after CCl_4_ injection (18 h after m17.ASC/Nluc injection), the mice were euthanized by cervical dislocation, and the lungs and liver were collected. The luciferase activity in lysed tissues was measured as described above.

### Therapeutic effect of PEG-MSCs in CCl_4_-induced acute liver failure mice

To establish a CCl_4_-induced acute liver failure model, 1 mL/kg CCl_4_ was intraperitoneally injected [31, 32] into FVB mice. The CCl_4_ dose was determined in preliminary experiments. Six hours after CCl_4_ injection, PBS, unmodified m17.ASC cells, or PEG_20k_-m17.ASC cells (1 × 10^6^ cells/mouse in 100 μL of PBS) were injected intravenously into mice under isoflurane anesthesia. Forty eight hours and 96 h after CCl_4_ injection (42 h and 90 h after m17.ASC injection), blood was collected from the mice under isoflurane anesthesia, and these were euthanized by cervical dislocation. Serum was collected as reported previously [29], and serum aspartate aminotransferase (AST) and alanine aminotransferase (ALT) levels were measured using the transaminase CII-test Wako kit according to the manufacturer’s protocol. For AST and ALT measurement after 48 h, 41 mice were used in the experiment (*n* = 13 CCl_4_-injured mice group versus *n* = 14 unmodified m17.ASC group versus *n* = 13 PEG_20k_-m17.ASC group). Two mice in the CCl_4_-injured mice group were excluded due to suspect of hemolysis of the blood samples during the blood sampling, and two mice in the PEG-_20k_-m17.ASC group were excluded due to death of the mice. For AST and ALT measurement after 96 h, 28 mice were used in the experiment (*n* = 4 normal mice group versus *n* = 8 CCl_4_-injured mice group versus *n* = 8 unmodified m17.ASC group versus *n* = 7 PEG_20k_-m17.ASC group). One mouse in the PEG-_20k_-m17.ASC group was excluded due to death of the mouse. In addition, 96 h after CCl_4_ injection (90 h after m17.ASC injection), the liver was collected and embedded in O.C.T. compound, frozen, and cut into 10 μm sections as described above. This experiment was conducted using 16 mice (*n* = 4 normal mice group versus *n* = 4 CCl_4_-injured mice group versus *n* = 4 unmodified m17.ASC group versus *n* = 4 PEG_20k_-m17.ASC group). The tissue slides were fixed with 4% paraformaldehyde solution and stained with hematoxylin and eosin according to a method published previously [33].

### Statistical analysis

Data were analyzed with the StatView software (SAS Institute, Inc., Cary, North Carolina, USA). A two-sided unpaired Student’s t-test was used to evaluate statistically significant differences between groups. Meanwhile, multiple group comparisons were performed using one-way ANOVA followed by Bonferroni/Dunnett’s test. Differences were considered statistically significant when *p* values were less than 0.05.

## Results

### PEG modification of the surface of MSCs

**Fig. 1:**
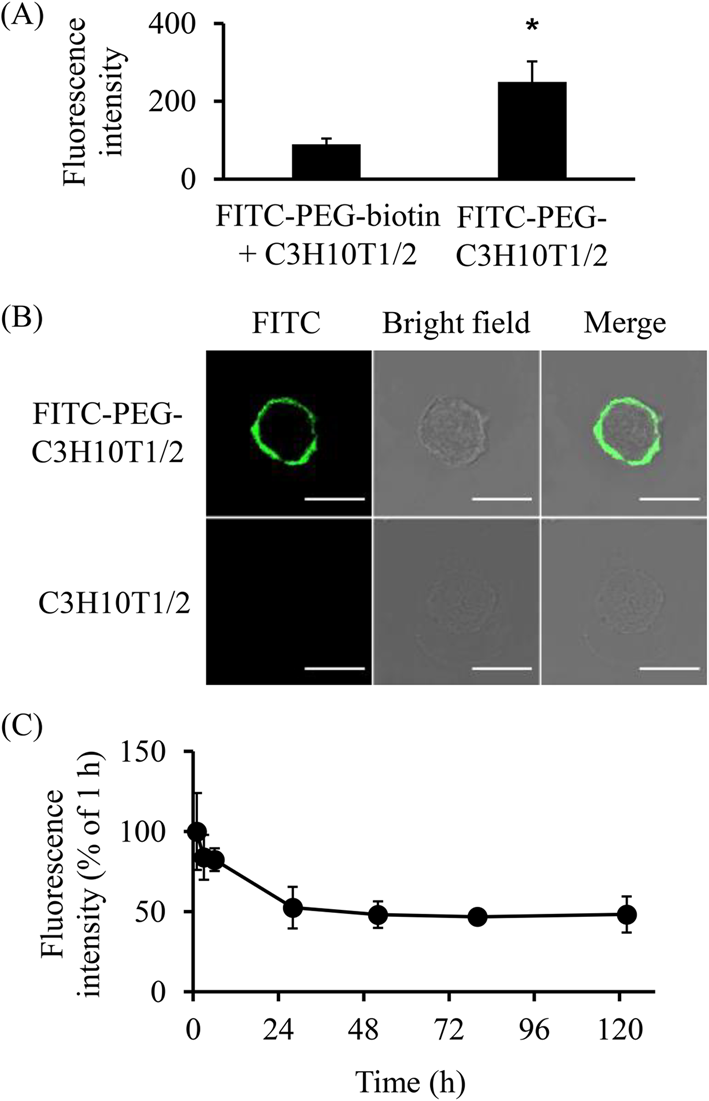
PEG modification to the surface of MSCs. **A** Fluorescence intensity of FITC-PEG-C3H10T1/2 cells. Streptavidin-modified C3H10T1/2 cells or unmodified C3H10T1/2 cells were incubated with FITC-PEG-biotin. The fluorescence intensity was measured using a microplate reader. The results are expressed as percentages of the value at 1 h. The error bars represent ± SD, *n* = 4, *p < 0.05 versus FITC-PEG-biotin + C3H10T1/2 group. **B** Confocal imaging of FITC-PEG-C3H10T1/2 cells. Streptavidin-modified C3H10T1/2 cells or unmodified C3H10T1/2 cells were incubated with FITC-PEG-biotin. The cells were cultured for 3 h and observed using a confocal laser scanning microscope (scale bars: 20 μm). **C** Retention time of PEG modification. Streptavidin-modified C3H10T1/2 cells were incubated with Alexa488-PEG-biotin, and the fluorescence intensity was measured using a microplate reader. The error bars represent ± SD, *n* = 4

**Fig. 2:**
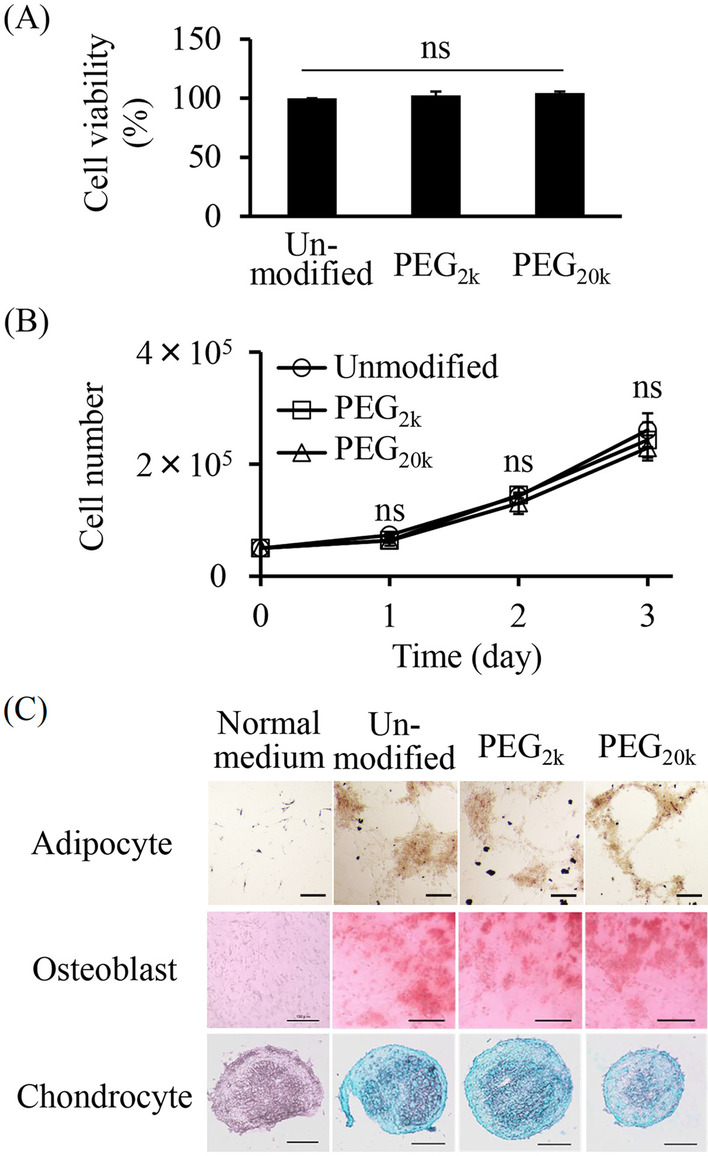
Characteristics of PEG-MSCs. **A** Cell viability of C3H10T1/2 cells after PEG modification. Streptavidin-modified C3H10T1/2 cells were incubated with PEG-biotin, and the cell viability was evaluated by trypan blue exclusion assay. The error bars represent ± SD, *n* = 4 (*ns*; not significant). **B** Proliferation of PEG-C3H10T1/2 cells. The number of PEG-C3H10T1/2 cells or unmodified C3H10T1/2 cells was measured daily by trypan blue exclusion assay. The error bars represent ± SD, *n* = 4 (*ns*; not significant). **C** Differentiation of PEG-C3H10T1/2 cells. Typical images of PEG-C3H10T1/2 cells and unmodified C3H10T1/2 cells differentiated into adipocytes, osteoblasts, or chondrocytes stained with oil red O, alizarin red S, and alcian blue, respectively (scale bars: 150 μm)

To evaluate the cell surface modification with PEG, we selected FITC- or Alexa488-labeled PEG-biotin. The fluorescence intensity of FITC in FITC-PEG-modified C3H10T1/2 (FITC-PEG-C3H10T1/2) was significantly higher than that in FITC-PEG-biotin-treated unmodified C3H10T1/2 (FITC-PEG-biotin + C3H10T1/2) (Fig. 1A). In addition, confocal microscopic images showed that FITC fluorescence signals were localized on the surface of C3H10T1/2 cells (Fig. 1B). These results indicate that the surface of C3H10T1/2 cells was modified successfully with FITC-PEG-biotin using the ABC method. The retention time of PEG modification was evaluated using Alexa488-PEG-biotin (Fig. 1C, Additional file 1: Fig. S1). The fluorescence intensity of Alexa488-PEG decreased gradually in 24 h. It remained at 50% of the initial value after 120 h of incubation. Although certain fluorescence signals were detected inside C3H10T1/2 cells at subsequent time-points, the signals remained on the cell surface even on day 5. Furthermore, the influence of PEG modification on the characteristics of C3H10T1/2 cells was evaluated. PEG modification negligibly affected the viability, proliferation, and differentiation of C3H10T1/2 cells (Fig. 2).

### Inhibition of cell adhesion by PEG modification

**Fig. 3:**
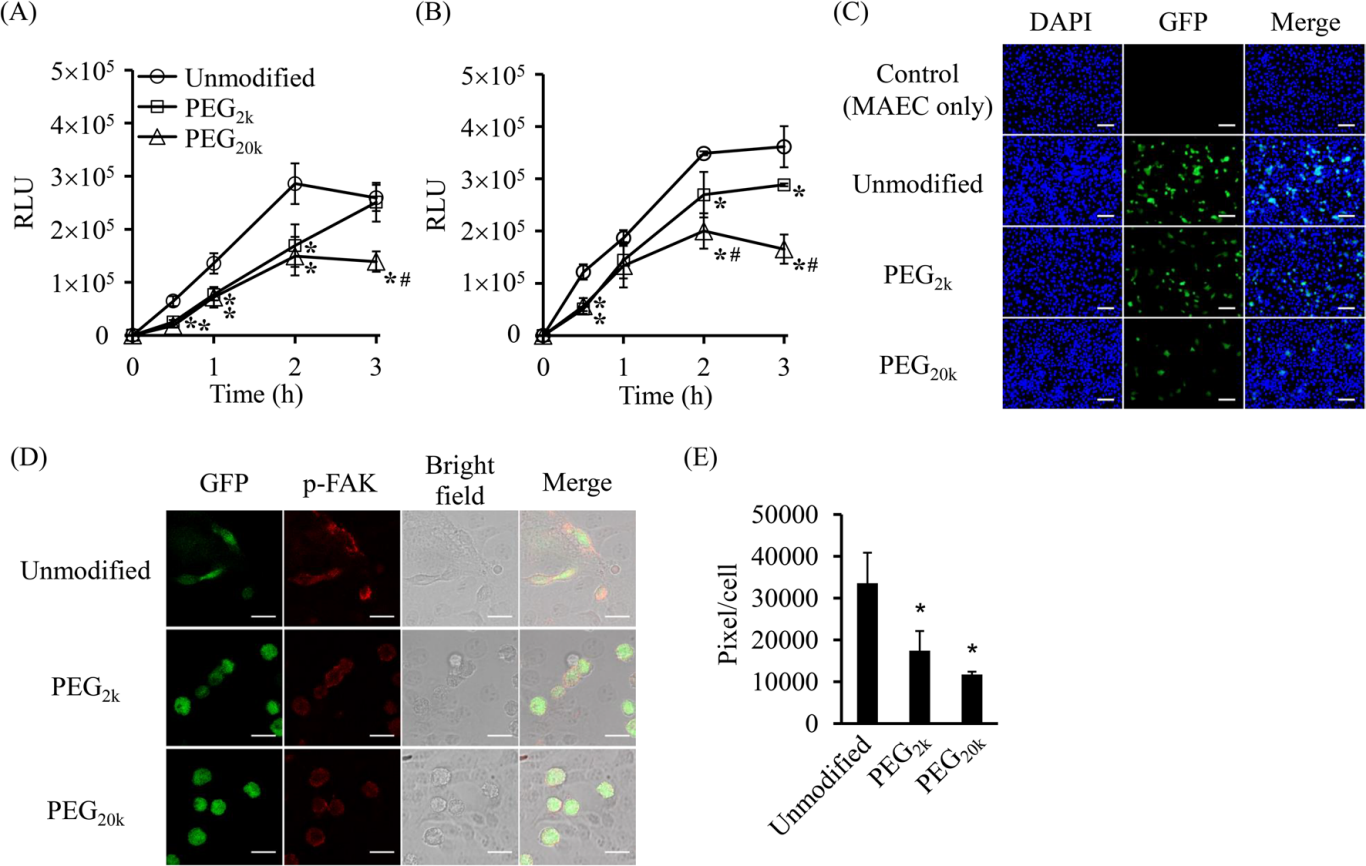
Inhibition of cell adhesion by PEG modification. **A** Number of C3H10T1/2 cells adhering to a culture plate. Unmodified or PEG-C3H10T1/2/Nluc cells were seeded onto a culture plate, and nonadherent cells were removed by PBS wash. The adhering cells were evaluated by measuring the luciferase activity. The error bars represent ± SD, *n* = 4, *p < 0.05 versus unmodified group, ^#^*p* < 0.05 versus PEG_2k_ group. **B, C** Adhesion of C3H10T1/2 cells to monolayered MAECs. Unmodified or PEG-C3H10T1/2 cells were seeded onto monolayered MAECs, and nonadherent C3H10T1/2 cells were removed by PBS wash. The adhering cells were evaluated by measuring the luciferase activity of C3H10T1/2/Nluc cells (**B**). Two hours after seeding, adhering C3H10T1/2/GFP cells were observed using a fluorescence microscope (**C**). The error bars represent ± SD, *n* = 4, *p < 0.05 versus unmodified group, ^#^*p* < 0.05 versus PEG_2k_ group (scale bars: 100 μm). **D, E** Immunofluorescence staining for p-FAK in C3H10T1/2/GFP cells. Unmodified or PEG-C3H10T1/2/GFP cells were seeded onto monolayered MAECs. Two hours after incubation, cells were stained and observed using a confocal laser scanning microscope (**D**). Furthermore, the fluorescence intensity from p-FAK was measured by ROI analysis (**E**). The error bars represent ± SD, *n* = 3, 15–20 cells analyzed per sample, *p < 0.05 versus unmodified group (scale bars: 20 μm)

The number of PEG-C3H10T1/2/Nluc cells was determined to evaluate the inhibitory effect of PEG modification on cell adhesion. The number of PEG_2k_- and PEG_20k_-C3H10T1/2/Nluc cells adhering to the culture plates was significantly lower than that of unmodified C3H10T1/2/Nluc cells (Fig. 3A). In addition, the number of PEG_2k_- and PEG_20k_-C3H10T1/2/Nluc cells adhering to MAECs was significantly lower than that of unmodified C3H10T1/2/Nluc cells (Fig. 3B). Furthermore, the inhibitory effect of PEG_20k_ modification was significantly higher than that of PEG_2k_ at 2 h and 3 h (Fig. 3B). Consistent with these results, microscopic observation showed that few PEG_20k_-C3H10T1/2/Nluc cells bound to MAECs (Fig. 3C). The mixture of PEG-biotin and C3H10T1/2/Nluc cells and the cell surface modification with only biotin or streptavidin negligibly affected the adhesion of C3H10T1/2 cells to MAECs (Additional file 1: Fig. S2). We then evaluated the activation of integrins by immunostaining to investigate whether PEG modification inhibits the interaction between cell adhesion molecules of C3H10T1/2 cells and MAECs. Integrins play important roles in cell adhesion. Immunostaining showed that PEG modification markedly suppressed the activation of FAK (a key mediator of intracellular signaling by integrins) at 2 h after seeding C3H10T1/2 cells. This indicates that PEG modification inhibited the activation of integrins in C3H10T1/2 cells (Fig. 3D, E). In addition, the PEG-modified cells were not spindle-shaped, indicating weak cell adhesion compared with unmodified C3H10T1/2 cells (Fig. 3D). Meanwhile, 12 h after seeding, no significant differences were observed in the adhesion of C3H10T1/2 cells to MAECs in any of the groups examined (Additional file 1: Fig. S3). These results indicate that PEG modification of the surface of MSCs using the ABC method temporally suppressed intercellular adhesion between MSCs and vascular endothelial cells. Because PEG_20k_ was better than PEG_2k_ in suppressing the adhesion of C3H10T1/2 cells to MAECs, PEG_20k_ was used for PEG modification in the following studies.

### Tissue distribution of PEG-MSCs after intravenous administration

**Fig. 4:**
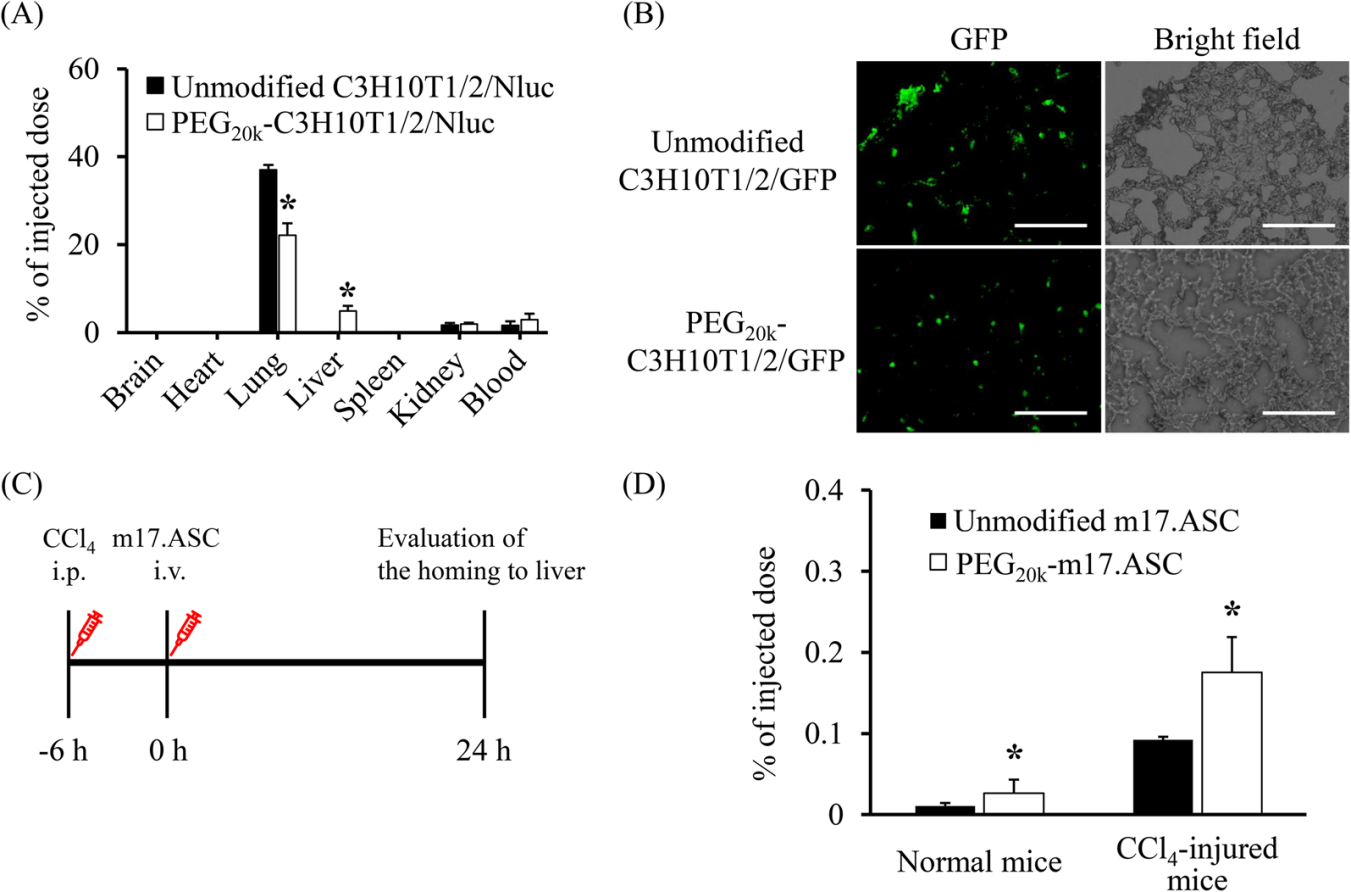
Inhibition of lung entrapment and improvement of homing efficiency of MSCs by PEG modification. **A** Tissue distribution of intravenously injected unmodified and PEG_20k_-C3H10T1/2 cells in normal mice. Unmodified or PEG_20k_-C3H10T1/2/Nluc cells were injected into the tail vein of C3H/He mice. One hour after injection, the organs and blood were collected, and the luciferase activity was measured. The error bars represent ± SD, *n* = 3, *p < 0.05 versus unmodified group. **B** Typical images of lung tissue section. Frozen lung sections were observed 1 h after injection of CFSE-labeled unmodified or PEG_20k_-C3H10T1/2 cells (scale bars: 100 μm). **C, D** Homing of m17.ASC cells to injured liver in CCl_4_-induced acute liver failure model mice. **C** Schematic image of experimental design. Schematic created with Microsoft PowerPoint. **D** The luciferase activity in the liver was measured 24 h after intravenous injection of unmodified or PEG_20k_-m17.ASC/Nluc cells to normal or CCl_4_-injured FVB mice. The error bars represent ± SD, *n* = 4, *p < 0.05 versus unmodified group

The tissue distribution of PEG-C3H10T1/2 cells in C3H/He mice was evaluated to determine the effect of PEG modification on lung entrapment of intravenously injected MSCs. PEG_20k_-C3H10T1/2/Nluc cells or unmodified C3H10T1/2/Nluc cells were injected into the tail vein of C3H/He mice. One hour after injection, the number of PEG_20k_-C3H10T1/2/Nluc cells in the lung was significantly lower than that of unmodified C3H10T1/2/Nluc cells (Fig. 4A). Moreover, the number of PEG_20k_-C3H10T1/2/Nluc cells in the liver was significantly higher than that of unmodified C3H10T1/2/Nluc cells. Consistent with this outcome, the number of CFSE-labeled PEG_20k_-C3H10T1/2 cells in lung tissue sections was lower than that of CFSE-labeled C3H10T1/2 cells (Fig. 4B). These results indicate that PEG_20k_ modification to the surface of MSCs using the ABC method suppressed the lung entrapment of intravenously administered MSCs by preventing intercellular adhesion between MSCs and vascular endothelial cells in the lung. Then, the homing efficiency of intravenously injected PEG-MSCs to inflammation sites was evaluated using a CCl_4_-induced acute liver failure model (which is commonly used to evaluate MSC homing [34–36]) and m17.ASC cells (which showed a high homing capability to an injured liver in a previous study [37]). Six hours after CCl_4_ injection, the serum AST and ALT levels in the CCl_4_-injured mice group were significantly higher than those in the vehicle-treated group (Additional file 1: Fig. S4). Based on these results, m17.ASC cells were injected 6 h after CCl_4_ injection, and their homing to the injured liver was evaluated 24 h after injection (Fig. 4C). This approach was adopted because certain studies reported that intravenously administered MSCs can be detected in the liver several hours after MSC administration [5, 35, 38]. The number of PEG_20k_-m17.ASC/Nluc cells in the injured liver was significantly higher than that in the unmodified m17.ASC/Nluc cells (Fig. 4D). These results indicate that PEG_20k_ modification to the surface of m17.ASC cells suppressed lung entrapment and improved homing to the injured liver after intravenous injection.

### Therapeutic effect of PEG-MSCs in acute liver failure model mice

**Fig. 5:**
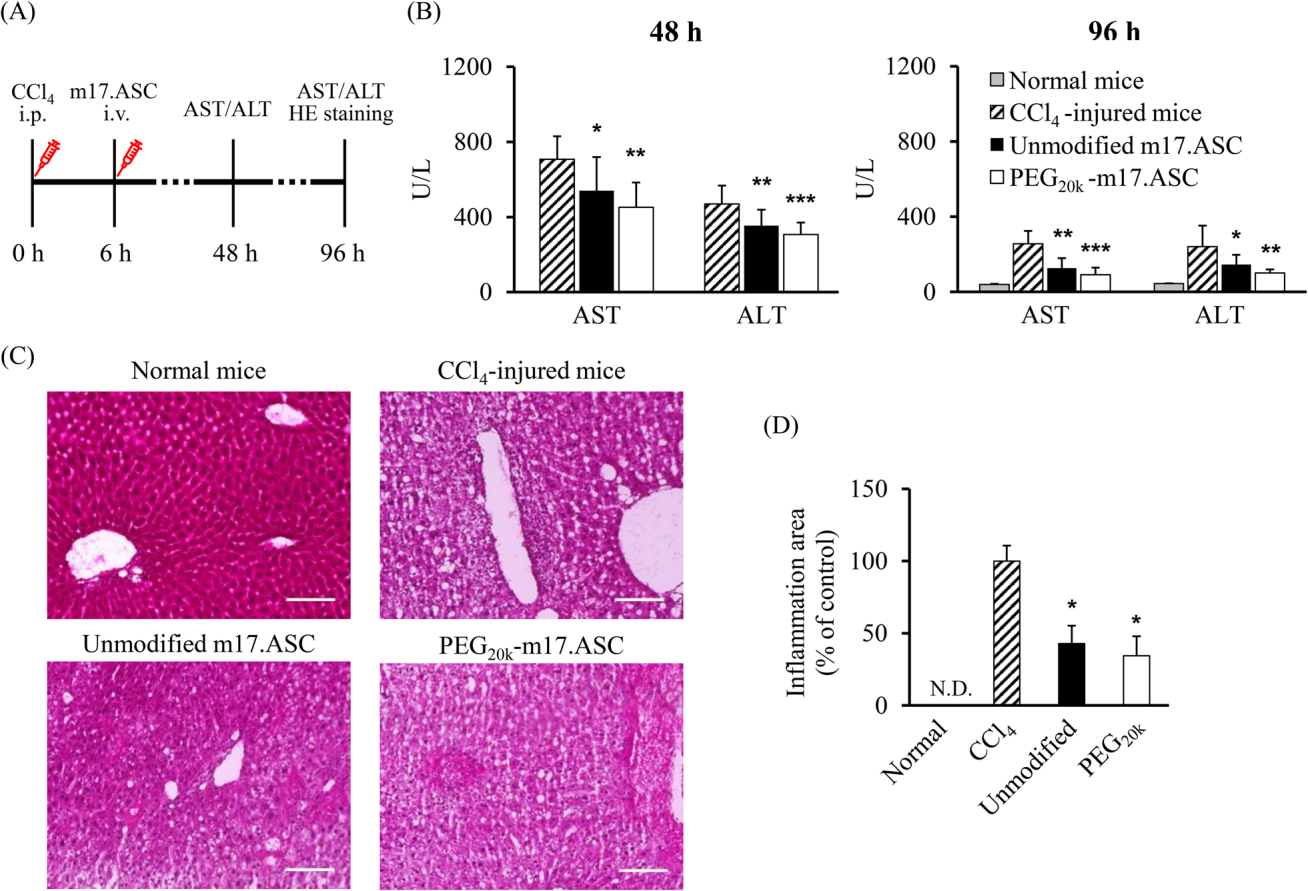
Therapeutic effect of PEG-MSCs in CCl_4_-induced acute liver failure model mice. **A** Schematic image of the experimental design. Schematic created with Microsoft PowerPoint. **B** Serum AST and ALT levels of acute liver failure model mice treated with PEG-m17.ASC cells. The serum AST and ALT levels were measured 48 h and 96 h after CCl_4_ injection (42 h and 90 h after m17.ASC injection). The normal mice group was administered neither CCl_4_ nor m17.ASC cells. The CCl_4_-injured mice group was administered only CCl_4_. The error bars represent ± SD, *n* = 4–14, *p < 0.05, **p < 0.01, ***p < 0.001 versus CCl_4_-injured mice group. **C, D** Anti-inflammatory effect of PEG_20k_-m17.ASC cells. Ninety six hours after CCl_4_ injection (90 h after m17.ASC injection), the liver section was stained with hematoxylin and eosin (**C**). Furthermore, the inflammation area accompanying the leucocyte infiltration was measured by ROI analysis (**D**). The normal mice group was administered neither CCl_4_ nor m17.ASC cells. The CCl_4_-injured mice group was administered only CCl_4_. The error bars represent ± SD, *n* = 4, *p < 0.05 versus CCl_4_-injured mice group (scale bars: 100 μm. *N.D.* not detected)

The therapeutic effect of intravenously injected PEG-m17.ASC cells was evaluated using the CCl_4_-induced acute liver failure model (Fig. 5A). Forty eight hours after CCl_4_ injection, the serum AST levels of the unmodified m17.ASC group and PEG_20k_-m17.ASC group were significantly lower (*p* < 0.05 and *p* < 0.01, respectively) than that of the CCl_4_-injured mice group (Fig. 5B). The serum ALT level was also significantly lower (*p* < 0.01, *p* < 0.001, respectively). Ninety six hours after CCl_4_ injection, the serum AST levels of the unmodified m17.ASC group and PEG_20k_-m17. ASC group were significantly lower (*p* < 0.01 and *p* < 0.001, respectively) than that of the CCl_4_-injured mice group. The serum ALT level was also significantly lower (*p* < 0.05, *p* < 0.01, respectively). Furthermore, leukocyte infiltration in the injured liver of the unmodified and PEG_20k_ groups was significantly lower than that in the CCl_4_-injured mice group (Fig. 5C, D). Overall, the PEG_20k_ group showed superior therapeutic effects compared with the unmodified group. These results indicate that PEG_20k_ modification to the surface of MSCs improved the homing efficiency to the inflammation sites and the therapeutic effect of intravenously administered MSCs.

## Discussion

Modifying cell surface properties to prevent lung entrapment of MSCs after intravenous injection can be a key technique for successful systemic MSC-based therapy. A few clinical studies have demonstrated that intravenous administration of MSCs exhibits effective therapeutic outcomes against diseases such as acute myocardial infarction, type 2 diabetes, diabetic nephropathy, rheumatoid arthritis, acute graft-versus-host disease, and coronavirus disease (COVID-19) [10, 11, 39, 40]. Although the therapeutic mechanism of MSCs is considered to involve paracrine effects and cell-to-cell interactions [6, 16, 17], the homing efficiency of intravenously administered MSCs to the target site is low. This indicates that MSCs do not fully exert their therapeutic potential. In the present study, the surfaces of MSCs were modified with PEG using the ABC method to improve the homing efficiency to the inflammation sites after intravenous injection. PEG modification temporarily suppressed the adhesion of C3H10T1/2 cells to MAECs and suppressed lung entrapment after intravenous injection of C3H10T1/2 cells. Furthermore, the homing efficiency and therapeutic effect of m17.ASC cells were improved by PEG modification. Therefore, PEG modification would be effective to improve the homing efficiency and therapeutic efficacy of MSCs by preventing lung entrapment.

Conventional cell surface modification with polymers (e.g., hydrophobic insertion of lipid conjugates and layer formation with charged polymers) causes cell membrane damage by long-term incubation with organic solvent-containing media [26]. Cell surface modification using the ABC method can rapidly and stably modify the cell surface with avidinated or biotinylated compounds under organic solvent-free conditions. These suitable conditions enable the modification of the surface of MSCs with minimum cytotoxicity [27]. In the present study, streptavidin-modified C3H10T1/2 cells were modified successfully with PEG-biotin after 10 min of incubation (Fig. 1A, B) without significant variations in the viability, proliferation, or differentiation capability (Fig. 2). In addition, PEG-C3H10T1/2 cells were differentiated into chondrocytes (Fig. 2C) which require cell–cell interaction in the differentiation process. This may be because the inhibitory effect of PEG modification on cell adhesion decreased with time in accordance with the internalization of PEG into the cytoplasm (Additional file 1: Fig. S1).

The molecular weight and other physicochemical properties of the compounds modified on the cell surface are important for the function of surface-modified cells. Teramura et al*.* reported that high molecular weight PEG (> 20 kDa; larger than certain cell adhesion molecules such as integrins) affected the cellular adhesion of PEG-modified cells. However, low molecular weight (2 kDa or 5 kDa) PEG had a negligible effect [25]. In the present study, 2 kDa PEG (PEG_2k_) and 20 kDa PEG (PEG_20k_) were used to investigate the effect of PEG chain length on the adhesion of C3H10T1/2 cells to endothelial cells. Since PEG_20k_ showed more pronounced effects than PEG_2k_ (Fig. 3A, B), we found that the PEG chain length was critical for cell adhesion. However, in our previous study, cell surface modification with Nluc (19 kDa) negligibly affected the cell adhesion of MSCs [27]. Yao et al. [41] reported that avidin (66 kDa) modification negligibly affected the cellular characteristics of MSCs. Similarly, streptavidin (55 kDa) modification did not significantly affect the cell adhesion of C3H10T1/2 cells (Additional file 1: Fig. S2)). These results indicate that PEG exhibits properties different from those of other macromolecular compounds on the surface of modified cells. PEG is widely used for surface modification of nanoparticles to prevent phagocytosis by mononuclear phagocytes [42]. PEG is a highly hydrophilic polymer, and PEG modification is considered to form an aqueous layer on the surface of nanoparticles [42]. Based on these characteristics, cell surface modification with PEG_20k_, but not with PEG_2k_, could efficiently inhibit the adhesion of C3H10T1/2 cells to endothelial cells by covering cell membrane proteins such as cell adhesion molecules.

Although many studies have shown the regulation of in vitro cell adhesion by cell surface modification, there are few reports demonstrating in vivo results on the regulation of cell adhesion by cell surface modification with polymers, particularly with PEG [21, 22, 25, 43–52]. In the present study, we demonstrated that PEG modification was effective for preventing lung entrapment of intravenously administered MSCs (Fig. 4B, C). However, the avoidance was incomplete. There are several factors that affect lung entrapment, including the size of MSCs [13], thrombus formation [34, 51–54], and intercellular adhesion between MSCs and endothelial cells in the lung [20–22]. It is necessary to control these factors simultaneously to achieve further reduction in lung entrapment. As shown in Fig. 4A, the total number of PEG_20k_-C3H10T1/2/Nluc cells (31.9%) detected in major organs and whole blood was lower than that of unmodified C3H10T1/2/Nluc cells (40.5%). This could be owing to the distribution of the cells to the unevaluated organs and tissues (e.g., brain, intestine, and muscle). On the other hand, some studies demonstrated that PEG modification improved the engraftment of islets by bypassing host immune systems [24, 55–62]. Therefore, improved survival of MSCs by PEG modification may increase the therapeutic effect after transplantation.

MSCs adhere to blood endothelial cells via various cell adhesion molecules [7]. In particular, integrins play an important role in cell adhesion between MSCs and blood endothelial cells in the lung vessel [22], and the PEG modification significantly inhibited the activation of integrins in C3H10T1/2 cells in vitro (Fig. 3D, E). These data suggest that the adhesion inhibitory effect by the PEG modification is due to the suppression of interaction between adhesion molecules of MSCs and endothelial cells. On the other hand, the in vivo adhesion between MSCs and endothelial cells takes place in a blood stream which is a dynamic environment. Some studies developed in vitro flow adhesion assay systems and demonstrated the strength of adhesion and steps of extravasation under physiological flow conditions [42, 59–62]. In the present study, PEG modification suppressed the adhesion of C3H10T1/2 cells to MAECs under an in vitro static condition and the entrapment of C3H10T1/2/Nluc cells in the lungs of mice (Figs. 3B, 4A). According to these results, the influence of the PEG modification on the lung entrapment of intravenously administered MSCs could be predicted by the simple and static experimental condition. Wang et al. [21] also succeeded in evaluating the influence of integrin antibodies for the lung entrapment of intravenously administered MSCs under an in vitro static experimental condition.

There are multiple processes in the homing of MSCs, such as chemokine-triggered activation, surface molecule-mediated rolling and adhesion to the endothelium, and transendothelial migration [13]. As shown in Fig. 4D, PEG modification significantly improved the homing efficiency of m17.ASC cells to the injured liver. This indicates that PEG modification negligibly affected the homing capability of MSCs to injured sites. In addition, the amount of PEG modified on the cell surface is important for the homing capability of MSCs. Although high-density or multiple layers coating of cell surface with polymers [63, 64] could inhibit the chemokine-triggered activation, in our previous study, cell surface modification with liposomes by the ABC method did not affect the migratory potency of MSCs due to low occupation (3.6% of the cell surface) [29]. Furthermore, the PEG modified on the cell surface decreased gradually and that the inhibitory effect of PEG on cell adhesion could weaken with time. The in vitro results (Fig. 1C) support this hypothesis. Furthermore, micropinocytosis (which is activated by chemokines [65]) may enhance PEG internalization, and the inhibiting effect may be reduced near the inflammation sites.

Considering the tissue distribution of PEG-modified MSCs, the distribution of PEG-modified MSCs to the liver dramatically increased in normal mice compared to that of unmodified MSCs (Fig. 4A), showing MSCs may be more likely to distribute to the liver, which is a large organ with rich blood flow and capillaries, after passing through the lung. In addition, the distribution of PEG-modified MSCs to the injured liver increased more (Fig. 4D), probably because of the homing ability of MSCs to injured sites. About the distribution to the other tissues, the remaining of PEG-modified MSCs in the blood increased 1 h after intravenous administration, but that to the other organs hardly changed (Fig. 4A), suggesting that PEG-modified MSCs escaped from lung entrapment, remained in the blood, and reaching mainly the liver. On the other hand, PEG-modified MSCs may not recognize the adhesion molecules in the injured liver. In this point as described above, the PEG modification on the cell surface gradually decreases, and the inhibitory effect of PEG on cell adhesion may become weakened with time. Thus, PEG modified on MSCs may temporarily suppress the lung entrapment, and then allow MSCs to interact with adhesion molecules in injured liver as PEG is gradually removed from cells.

Many studies have reported that a majority of intravenously administered MSCs are entrapped in the lung [13]. However, few attempts have been undertaken to prevent this entrapment and improve delivery efficiency to target sites [21, 22]. It is becoming increasingly evident that the main therapeutic mechanism of intravenously injected MSCs is paracrine effects or apoptosis in MSCs rather than differentiation into damaged cells [10, 66, 67]. Certain studies reported that although intravenously injected MSCs were distributed mainly in the lung, the inflammatory reaction was alleviated in liver failure model mice [15], drug-induced nephropathy model mice [68], and pancreatitis model mice [69]. These studies indicated that secretomes from MSCs in the lungs show immune regulatory and tissue repair effects. However, certain groups recently reported that direct cell-to-cell interactions at the inflammation site are also important for immune regulatory and tissue repair effects of MSCs [17, 18]. Furthermore, although over 1000 clinical trials relevant to MSCs are registered on FDA.gov, many trials in large stages showed negligible therapeutic effect [11]. Furthermore, no MSC product was approved by the Food and Drug Administration (FDA) as of September 2021. In the present study, the improved homing efficiency demonstrated the higher therapeutic efficiency of intravenously administered m17.ASC cells. This indicates the importance of regulating the biodistribution of intravenously administered MSCs to achieve effective MSC-based therapy.

## Conclusion

MSCs were successfully modified with PEG using the ABC method without cell damage, and PEG modification prevented lung entrapment of intravenously administered MSCs. Furthermore, PEG modification improved the homing efficiency to the injured liver of CCl_4_-induced acute liver failure model mice, and intravenously injected PEG-MSCs suppressed serum transaminase levels and leukocyte infiltration in the injured liver. These results indicate that cell surface modification with PEG may be an effective engineering procedure for effective MSC-based therapy.

### Supplementary Information

**Additional file 1**. Supplementary figures.

## Data Availability

The dataset supporting the conclusions of this article is included within the article and its additional file.

## Abbreviations

ABC: Avidin–biotin complex
ALT: Alanine aminotransferase
AST: Aspartate aminotransferase
Nluc: NanoLuc luciferase
C3H10T1/2: Murine mesenchymal stem cell line
CCl_4_: Carbon tetrachloride
CFSE: Carboxyfluorescein diacetate succinimidyl ester
COVID-19: Coronavirus disease
FBS: Fetal bovine serum
HGF: Hepatocyte growth factor
MAECs: Mouse aortic endothelial cells
MSCs: Mesenchymal stem/stromal cells
m17.ASC: Murine adipose-derived mesenchymal stem cell line
PEG: Polyethylene glycol
p-FAK: Phospho-focal adhesion kinase
ROI: Region of interest
SPF: Specific pathogen-free
TRITC: Tetramethylrhodamine isothiocyanate
