# Supplementary material for: Efficient delivery of mesenchymal stem/stromal cells to injured liver by surface PEGylation
Source: Stem Cell Res Ther. 2023 Aug 22;14:216. doi: 10.1186/s13287-023-03446-w (PMC10464485; doi:10.1186/s13287-023-03446-w)
Supplement: Supplementary file 1 — Additional file 1. Supplementary figures. [file 13287_2023_3446_MOESM1_ESM.docx]

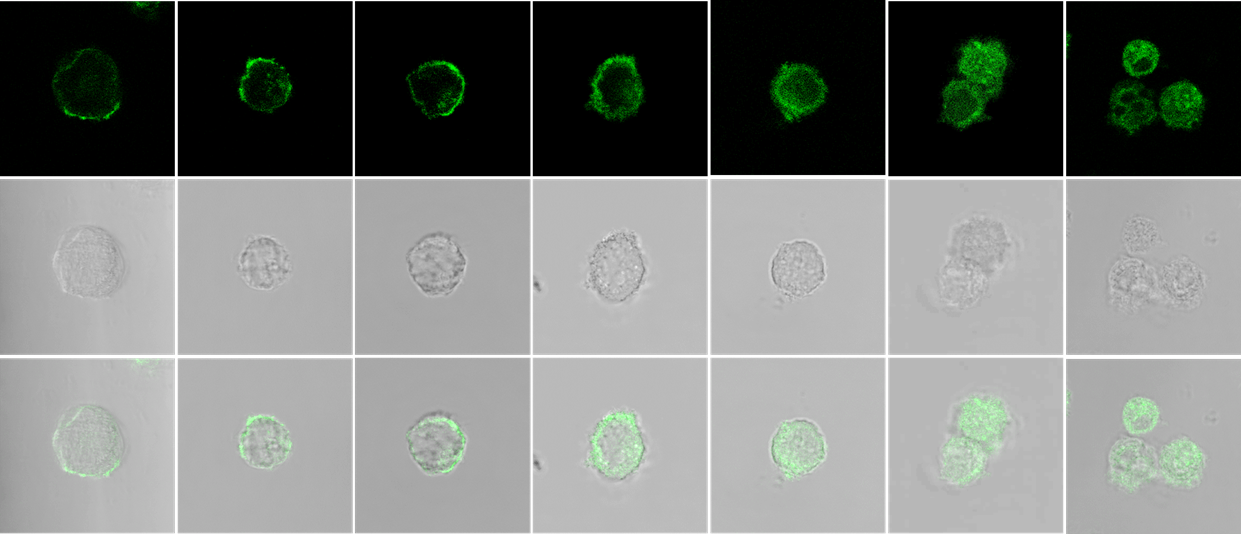


1 h

3 h

6 h

1 d

2 d

3 d

5 d

GFP

Bright field

Merge

**Fig. S1.** Confocal imaging of Alexa488-PEG-modified C3H10T1/2 cells. Alexa488-PEG-modified C3H10T1/2 cells were cultured on Nunc Lab-Tek II Chambered Coverglasses and observed using a confocal laser scanning microscope (scale bars: 20 μm).


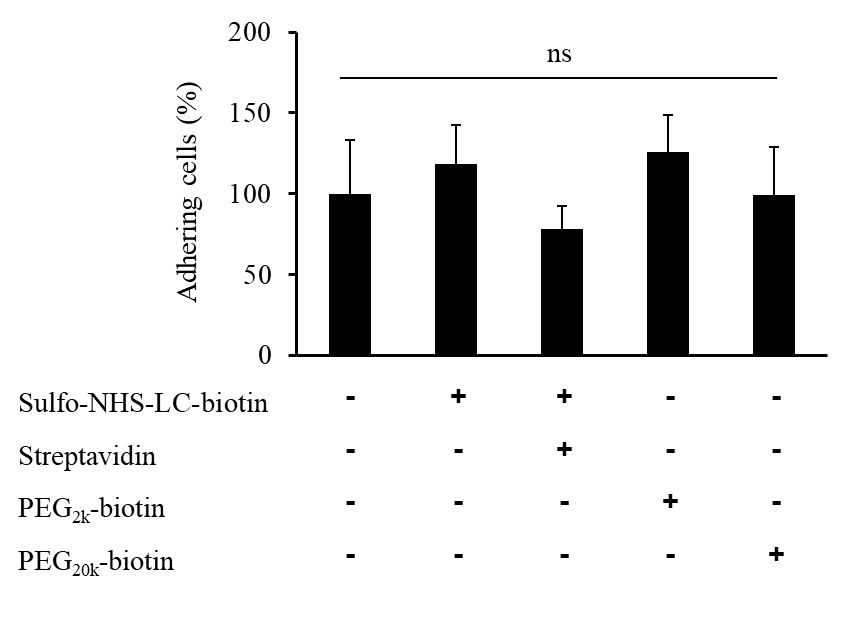


**Fig. S2.** Influence of biotin- or streptavidin-modification on cell adhesion. Biotin- or streptavidin-modified C3H10T1/2/Nluc cells or, PEG-biotin-treated unmodified C3H10T1/2/Nluc cells were seeded onto a monolayered MAECs. Two hours after incubation, nonadherent C3H10T1/2/Nluc cells were removed by PBS wash and the luciferase activity was measured. The error bars represent ± SD, n = 4 (ns; not significant).


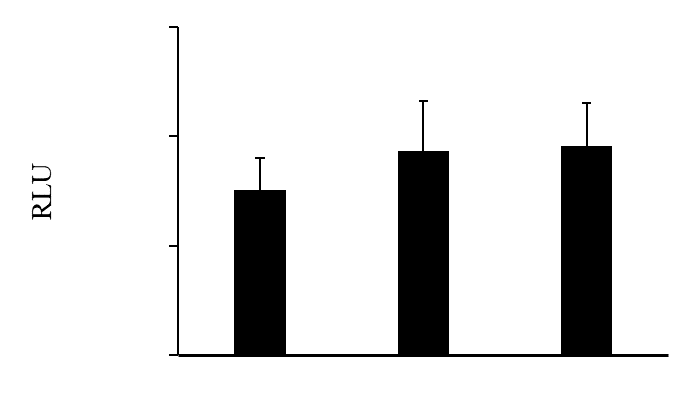


ns

Unmodified

PEG_2k_

PEG_20k_

6×10^5^

4×10^5^

3×10^5^

0

**Fig. S3.** Number of PEG-C3H10T1/2 cells adhering to MAECs at 12 h after seeding. PEG2k-C3H10T1/2 cells, PEG20k-C3H10T1/2/Nluc cells, and unmodified C3H10T1/2/Nluc were seeded onto a monolayered MAECs. Twelve hours after seeding, nonadherent C3H10T1/2/Nluc cells were removed by PBS wash, and the luciferase activity was measured. The error bars represent ± SD, n = 4 (ns; not significant).

**_*_**

**_*_**


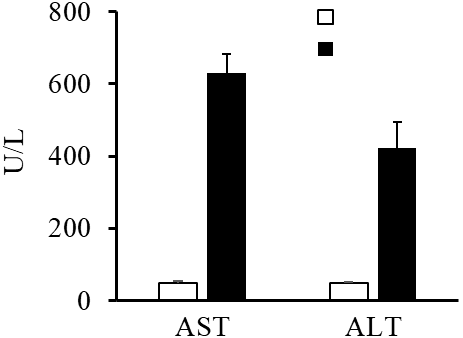


Vehicle

CCl_4_

Vehicle

**Fig. S4.** Serum AST and ALT levels of CCl_4_-induced acute liver failure model mice. CCl_4_ (1.5 mL/kg) was intraperitoneally injected to FVB mice, at 6 h after injection, the serum AST and ALT levels were measured. The error bars represent ± SD, n = 4, ^*^p < 0.05 versus vehicle group.
